# A Case of Fetus Papyraceus

**Published:** 1889-08

**Authors:** E. W. Mulligan

**Affiliations:** Rochester, N. Y.


### Clinical Reports.

### *A CASE OF FETUS PAPYRACEUS.*[1]

By E. W. MULLIGAN, M. D., Rochester, N. Y.

NOVEMBER 21, 1888, called to see Mrs. L———. She was having abdominal pains, intermittent in character, coming about once in a half-hour. She told me that she menstruated last the latter part of May, 1888. This would show pregnancy of six months. I concluded that she would be delivered in due time, that she was mistaken regarding time of last menstruation, as her abdomen was very large, and, as she had made no progress, I left her, telling the nurse to send for me when pains became more severe and rapid. I did not see her again until February 28, 1889, when I found her *again* in labor, and this time her abdomen was much smaller than when I saw her three months before. But now there was no doubt about it; the os was well dilated, and head presented. In about an hour she was delivered of a ten-pound boy, and, immediately following this, a dead fetus, well preserved. The dead child was flattened out, so that it took up but little space. It was, I should judge, a six-months' fetus. The cord was slightly attached to the border of the placenta. I now made enquiry concerning her sickness when I last saw her, three months previously. She said that soon after I left her, the bag of waters broke, and almost a pailful of clear fluid escaped. After this, the pains subsided, and she went about her duties as usual. It is plain to be seen that this was a case of "the survival of the fittest." At my first visit, both children were in all probability alive, but, after the rupture of the sac and escape of the amniotic liquid, one died. If this had not occurred, we would have had a miscarriage, and probably lost both. This is of interest to show that a dead fetus can be retained in the uterus for three months, and no harm come to the mother. Lusk describes this condition of things, and says that the dead child may be aborted, while the living fetus advances to full term of gestation.

### DOUBLE VAGINA AND UTERUS.

I also wish to report a case of double vagina and uterus occurring in the practice of my associate, Dr. Jonas Jones. I saw and examined the case with Dr. Jones. The patient, a well-developed young woman, aged twenty-five years, married two years, without any children. The

1. Read at the meeting of the Monroe County Medical Society, May 29, 1889.

external genitals are normal. The vaginas are situated side by side, the opening to the left one being a little above the right. They appear to be about the same size, but the right one having been used, received the speculum more easily. Each uterus is two and a half inches in depth, and the os normal in both. She has had two miscarriages in past three years, miscarrying each time when two months pregnant. Skene describes a similar case in his work.

